# Supplementary material for: Minimally disruptive optical control of protein tyrosine phosphatase 1B
Source: Nat Commun. 2020 Feb 7;11:788. doi: 10.1038/s41467-020-14567-8 (PMC7005756; doi:10.1038/s41467-020-14567-8)
Supplement: Supplementary file 10 — Description of Additional Supplementary Files [file 41467_2020_14567_MOESM10_ESM.pdf]

Title: Supplementary Data 1.
Description: Analysis of Photoswitching. This table provides estimates of dynamic range, including error and sample sizes, for the activity of PTP-LOV2 chimeras on 4MUP, pNPP, and a phosphopeptide substrate.

Title: Supplementary Data 2.
Description: Analysis of kinetics. This table provides initial rates of pNPP hydrolysis, including error and sample sizes, used to construct Michaelis-Menten curves.

Title: Supplementary Data 3.
Description: Analysis of reversibility. This table provides initial rates of pNPP hydrolysis, including error and sample sizes, used to examine the reversibility of photomodulation.

Title: Supplementary Data 4.
Description: Circular dichroism spectroscopy. This table provides CD-based estimates of light-induced changes in α-helical content, including error and sample sizes, used to construct Figures 2c and 2g.

Title: Supplementary Data 5.
Description: Fluorescence spectroscopy. This table provides fluorescence-based measurements of tryptophan fluorescence, including error and sample sizes, used to construct Figure 2g.

Title: Supplementary Data 6.
Description: In vitro analysis of FRET-based biosensor. This table provides discrete measurements of donor/acceptor emission ratio, including error and sample sizes, used to construct Figures 4b and 4c.

Title: Supplementary Data 7.
Description: Whole-cell imaging studies. This table provides live-cell FRET measurements used to construct Figure 4c.

Title: Supplementary Data 8.
Description: Enzyme-linked immunosorbent assay (ELISA). This table provides ELISA-based measurements of insulin receptor phosphorylation.
