# Supplementary material for: Minimally disruptive optical control of protein tyrosine phosphatase 1B
Source: Nat Commun. 2020 Feb 7;11:788. doi: 10.1038/s41467-020-14567-8 (PMC7005756; doi:10.1038/s41467-020-14567-8)
Supplement: Supplementary file 11 — Reporting Summary [file 41467_2020_14567_MOESM11_ESM.pdf]

nature research

Corresponding author(s): Jerome M. Fox

Last updated by author(s): 01/11/2020

Reporting Summary

Nature Research wishes to improve the reproducibility of the work that we publish. This form provides structure for consistency and transparency in reporting. For further information on Nature Research policies, see Authors & Referees and the Editorial Policy Checklist .

Statistics

For all statistical analyses, confirm that the following items are present in the figure legend, table legend, main text, or Methods section.

n/a	Confirmed	
[ ]	[x]	The exact sample size (*n*) for each experimental group/condition, given as a discrete number and unit of measurement
[ ]	[x]	A statement on whether measurements were taken from distinct samples or whether the same sample was measured repeatedly
[ ]	[x]	The statistical test(s) used AND whether they are one- or two-sided<br>*Only common tests should be described solely by name; describe more complex techniques in the Methods section.*
[x]	[ ]	A description of all covariates tested
[ ]	[x]	A description of any assumptions or corrections, such as tests of normality and adjustment for multiple comparisons
[ ]	[x]	A full description of the statistical parameters including central tendency (e.g. means) or other basic estimates (e.g. regression coefficient) AND variation (e.g. standard deviation) or associated estimates of uncertainty (e.g. confidence intervals)
[ ]	[x]	For null hypothesis testing, the test statistic (e.g. *F*, *t*, *r*) with confidence intervals, effect sizes, degrees of freedom and *P* value noted<br>*Give P values as exact values whenever suitable.*
[x]	[ ]	For Bayesian analysis, information on the choice of priors and Markov chain Monte Carlo settings
[x]	[ ]	For hierarchical and complex designs, identification of the appropriate level for tests and full reporting of outcomes
[x]	[ ]	Estimates of effect sizes (e.g. Cohen's *d*, Pearson's *r*), indicating how they were calculated

Our web collection on statistics for biologists contains articles on many of the points above.

Software and code

Policy information about availability of computer code

Data collection	NIS-Elements, SoftMax Pro, Chirascan, Unicorn, Image Lab
Data analysis	NIS-Elements, Datagraph, ApE, Chirascan, EMBOSS Needle, Image Lab, PyMOL, Excel

For manuscripts utilizing custom algorithms or software that are central to the research but not yet described in published literature, software must be made available to editors/reviewers. We strongly encourage code deposition in a community repository (e.g. GitHub). See the Nature Research guidelines for submitting code & software for further information.

Data

Policy information about availability of data

All manuscripts must include a data availability statement. This statement should provide the following information, where applicable:

- Accession codes, unique identifiers, or web links for publicly available datasets
- A list of figures that have associated raw data
- A description of any restrictions on data availability

The source data underlying Figures 1c, 1e, 1f, 2a, 2b, 2c, 2d, 2e, 2g, 3b, 3c, 4b, 4c, 4e, and 4g and Supplementary Figures 1b, 1c, 4a, 4c, 5b, 5c, 5d, 6b, 7b, 8c, 8d, 9c, 9d, 10c, 10d, 11a, 11c, 12a, and 12b are provided as a Source Data file; this data includes samples sizes for each dataset. For clarity, Supplementary Tables 3-10 group data by type. The crystal structure determined in this study is available from the RCSB Protein Data Bank (PDB entry 6ntp [https://www.rcsb.org/structure/6ntp]). Table 1 provides the refinement statistics for this structure. Plasmids harboring important genes used in this study are available from Addgene: LOV2 (pTriEx-PA-Rac1, #22024,) full-length PTP1B (pGEX-2T-PTP1B , #8602), and biosensor (Kras-Src FRET biosensor, #78302), All other raw data not included in the manuscript are available from the corresponding author upon request.

Field-specific reporting

Please select the one below that is the best fit for your research. If you are not sure, read the appropriate sections before making your selection.

- [x] Life sciences
- [ ] Behavioural & social sciences
- [ ] Ecological, evolutionary & environmental sciences

For a reference copy of the document with all sections, see nature.com/documents/nr-reporting-summary-flat.pdf

Life sciences study design

All studies must disclose on these points even when the disclosure is negative.

Sample size	For our analysis of photoswitching, we used discrete measurements to estimate initial rates and, thus, to calculate DR (i.e., Vo-dark/Vo-light). We minimized error in our measurements of photoswitching with four precautions: (i) We used concentrations of enzyme and substrate that sustained initial reaction rates for 42 minutes, a length of time that minimizes the disruption of 1-min breaks required to measure product formation. (ii) For each construct in each plate, we prepared two sets of three compositionally identical, yet differentially positioned wells; this arrangement minimizes potential contributions from nonuniform illumination. (iii) For each construct at each illumination condition, we repeated the assay at least three times, collecting a total six estimates of initial rate, each based on measurements from three wells. We performed all other kinetic experiments with at least three independent biological replicates (i.e., protein samples). For whole-cell irradiation studies, we collected data from 11 individual cells for each chimera; for localized activation studies, we collected data from 6 individual cells for each chimera; and for our enzyme-linked immunosorbent assay (ELISA), we carried out analyses of three biological replicates. For all studies of mammalian cells, we analyzed cells on at least two separate days.
Data exclusions	For our analysis of photoswitching, we established a control range: When wells containing wild-type PTP1B showed a 10% difference in activity between the two plates, we discarded data from both plates (i.e., we assumed that differences in activity between the two plates were not caused by the presence or absence of light). For our whole-cell activation study, we analyzed the interquartile average of 11 experiments (i.e., 11 cells) performed with each chimera; final averages and standard errors, thus, reflect measurements of n = 5 cells.
Replication	We collected data with technical and biological replicates (n = 3 to 18) on different days. Measurements from different days closely matched one another.
Randomization	Randomization is not relevant to this study.
Blinding	Blinding is not relevant to this study.

Reporting for specific materials, systems and methods

We require information from authors about some types of materials, experimental systems and methods used in many studies. Here, indicate whether each material, system or method listed is relevant to your study. If you are not sure if a list item applies to your research, read the appropriate section before selecting a response.

Materials & experimental systems

n/a	Involved in the study	
[ ]	[x]	Antibodies
[ ]	[x]	Eukaryotic cell lines
[x]	[ ]	Palaeontology
[x]	[ ]	Animals and other organisms
[x]	[ ]	Human research participants
[x]	[ ]	Clinical data

Methods

n/a	Involved in the study	
[x]	[ ]	ChIP-seq
[x]	[ ]	Flow cytometry
[x]	[ ]	MRI-based neuroimaging

Antibodies

Antibodies used	We used an antibody-coated 96-well plate (insulin receptor β rabbit mAb coated microwells; #18872) and two free antibodies: a detection antibody (phospho-tyrosine mouse detection mAb; #12982) and a secondary detection antibody (anti-mouse IgG, HRP-linked antibody; #13304), all supplied by Cell Signaling Technologies.
Validation	Cell Signaling Technology uses a variety of methods for application-specific validation antibodies. These methods, which are detailed online [https://www.cellsignal.com/contents/our-approach/cst-antibody-validation-principles/ourapproach-validation-principles], include analyses of cell lines with target expression levels (e.g., CHO-IR/IRS-1 cells with insulin stimulates tyrosine phosphorylation of Insulin Receptor β).

Eukaryotic cell lines

Policy information about cell lines

Cell line source(s)	COS-7 (ATCC CRL-1651) and HEK293T/17 cells (ATCC CRL-11268)
Authentication	Morphology check by microscope (mammalian cells).
Mycoplasma contamination	The cells used in this study tested negative for mycoplasma contamination.
Commonly misidentified lines (See ICLAC register)	Commonly misidentified lines were not used in this study.
